# Colonocyte-derived lactate promotes *E. coli* fitness in the context of inflammation-associated gut microbiota dysbiosis

**DOI:** 10.1186/s40168-022-01389-7

**Published:** 2022-11-26

**Authors:** Savannah J. Taylor, Maria G. Winter, Caroline C. Gillis, Laice Alves da Silva, Amanda L. Dobbins, Matthew K. Muramatsu, Angel G. Jimenez, Rachael B. Chanin, Luisella Spiga, Ernesto M. Llano, Vivian K. Rojas, Jiwoong Kim, Renato L. Santos, Wenhan Zhu, Sebastian E. Winter

**Affiliations:** 1 Department of Microbiology, University of Texas Southwestern Medical Center, Dallas, TX USA; 2 Present Address: Department of Internal Medicine, Division of Infectious Diseases, UC Davis Health, Davis, CA 95616 USA; 3 Present Address: Novome Biotechnologies, South San Francisco, CA 94080 USA; 4 Departamento de Clínica e Cirurgia Veterinárias, Escola de Veterinária, Universidade Federal de Minas Gerais, Belo Horizonte, MG 31270 Brazil; 5 Present Address: Infectious Diseases, Genentech, South San Francisco, CA 94080 USA; 6 Present Address: Department of Medicine, Hematology, Blood and Marrow Transplantation, Stanford University, Stanford, CA USA; 7 Department of Pathology, Microbiology, and Immunology, Vanderbilt University Medical Center, Nashville, USA; 8 Department of Population and Data Sciences, UT Southwestern Medical Center, Dallas, TX 75390 USA

**Keywords:** Host-microbe interactions, Lactate metabolism, Gut inflammation

## Abstract

**Background:**

Intestinal inflammation disrupts the microbiota composition leading to an expansion of Enterobacteriaceae family members (dysbiosis). Associated with this shift in microbiota composition is a profound change in the metabolic landscape of the intestine. It is unclear how changes in metabolite availability during gut inflammation impact microbial and host physiology.

**Results:**

We investigated microbial and host lactate metabolism in murine models of infectious and non-infectious colitis. During inflammation-associated dysbiosis, lactate levels in the gut lumen increased. The disease-associated spike in lactate availability was significantly reduced in mice lacking the lactate dehydrogenase A subunit in intestinal epithelial cells. Commensal *E. coli* and pathogenic *Salmonella*, representative Enterobacteriaceae family members, utilized lactate via the respiratory L-lactate dehydrogenase LldD to increase fitness. Furthermore, mice lacking the lactate dehydrogenase A subunit in intestinal epithelial cells exhibited lower levels of inflammation in a model of non-infectious colitis.

**Conclusions:**

The release of lactate by intestinal epithelial cells during gut inflammation impacts the metabolism of gut-associated microbial communities. These findings suggest that during intestinal inflammation and dysbiosis, changes in metabolite availability can perpetuate colitis-associated disturbances of microbiota composition.

Video Abstract

**Supplementary Information:**

The online version contains supplementary material available at 10.1186/s40168-022-01389-7.

## Background

Host-associated microbial communities fulfill beneficial functions, such as educating the immune system and facilitating metabolism. These nuanced and multifaceted interactions present many opportunities for imbalance and disturbances, and commensal microbes are involved in a number of disease processes [1–4]. Experimental perturbations of complex systems, such as the interaction of the gut microbiota with its host, provide unique opportunities to gain insights into the underlying molecular mechanisms [5, 6]. During episodes of gastrointestinal inflammation, the composition of the gut microbiota changes at the phylum level (dysbiosis). The abundance of Gammaproteobacteria, in particular Enterobacteriaceae family members, increases while the relative abundance of obligate anaerobic bacteria declines [7–10]. This unbalanced microbiota can worsen colitis [11, 12], for example through reduced production of immunomodulatory bile acids [13]. Dysbiosis also increases the risk for the development of colitis-associated colorectal cancer [14]. Conversely, preventing the bloom of Enterobacteriaceae family members alleviates mucosal inflammation and decreases the risk of inflammation-associated colorectal cancer [15, 16]. A better understanding of the dynamics of functional host-microbe interactions may hold great promise for developing rationally designed, microbiota-targeting intervention strategies [17–21].

One key driver of inflammation-associated changes in the gut microbiota is the perturbation of metabolic interactions. In IBD patients, bacterial and host metabolic activities are disturbed, and disease-specific metabolites have been identified [22]. For example, increased availability of oxygen and the release of byproducts of inflammatory reactive oxygen and nitrogen metabolism enhance growth of Enterobacteriaceae in mouse models [23, 24]. An overabundance of *N*-acylethanolamines in IBD patients inhibits the growth of obligate beneficial microbes while promoting the growth of potentially harmful microbes such as Enterobacteriaceae and Enterococcaceae [25]. These studies highlight the importance of metabolism-based host-microbe interactions.

**Fig. 1:**
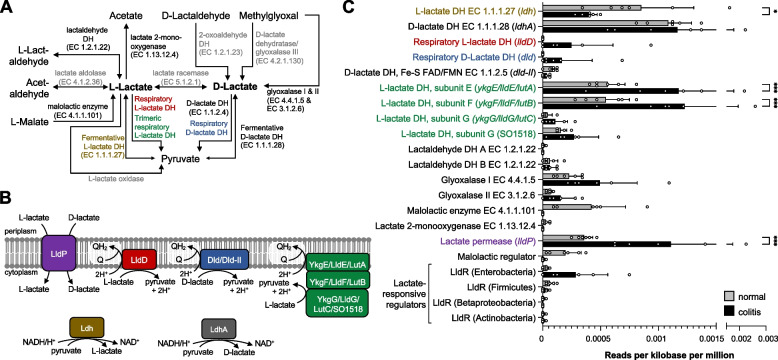
Microbiota coding capacity for lactate utilizing genes in murine colitis model. **A** Schematic representation of D- and L-lactate metabolism. **B** Graphical representation of bacterial lactate utilizing enzymes. Lactate permease (LldP) facilitates the transport of both L- and D-lactate. The three membrane-bound respiratory lactate dehydrogenases, L-lactate dehydrogenase (LldD), D-lactate dehydrogenase (Dld), and the trimeric L-lactate dehydrogenase (YkgEFG/ LldDEFG/ LutABC), all convert pyruvate to lactate while contributing electrons to the quinone pool. Both fermentative L-lactate dehydrogenase (Ldh) and fermentative D-lactate dehydrogenase (LdhA) are cytoplasmic and catalyze conversion of pyruvate to L- and D-lactate, respectively. **C** Microbiota coding capacity for pathways involved in microbial lactate metabolism in a murine colitis model. Metagenomics was performed on a previously described dataset (Hughes et al., 2017; ENA accession number PRJEB15095). The cecal microbiota of mice treated with 3% DSS in their drinking water (colitis; black bars) were compared to mock-treated mice (gray bars). The normalized number of reads for lactate-related functions in the KEGG orthology database for each mouse is plotted. Columns represent mean and error bars represent standard deviation. Significance was determined using a 2-way ANOVA with Sidak’s multiple comparisons test. *, *p*<0.05; ***, *p*<0.001

Despite the diversity of metabolic reactions that exist in nature, some key pathways exist in virtually every cell, such as the production or consumption of lactate (Fig. 1A). Lactate exists as two enantiomers, D- and L-lactate. Organisms from all domains of life are known to maintain redox balance in the absence of respiration by converting pyruvate to lactate, a reaction catalyzed by stereospecific, NAD-dependent, lactate dehydrogenases (DH) located in the cytosol [26, 27]. Bacteria and archaea produce fermentative D- and L-lactate DH under anaerobic conditions in the absence of exogenous electron acceptors [28, 29]. Mammalian cells primarily produce L-lactate when oxidative phosphorylation cannot effectively fulfill energy requirements of the cell. For example, skeletal muscle cells convert pyruvate to L-lactate when oxygenation is insufficient [27]. Tumor cells typically perform glycolysis coupled to L-lactate fermentation even in the presence of oxygen (Warburg effect) [30]. Other metabolic pathways that generate D- or L-lactate involve the degradation of malate, lactaldehyde, and methylglyoxal (methylglyoxal pathway) (Fig. 1A) [28, 31]. Microbial lactate degradation either involves conversion to pyruvate by membrane-associated, respiratory lactate DHs, conversion to acetate and CO_2_ by lactate 2-monooxygenase, conversion to pyruvate and hydrogen peroxide by lactate oxidase, or conversion to acetaldehyde and formate by lactate aldolase (Fig. 1A) [29, 32, 33]. In contrast to fermentative lactate DHs, bacterial respiratory lactate DHs donate electrons generated by the oxidation of lactate to the membrane-bound quinone pool, coupling lactate degradation to an electron transport chain (Fig. 1B) [29]. Despite its central importance in microbial and host physiology, little is known about lactate metabolism of the gut microbiota in the context of inflammation.

In a previous study, we used comparative metagenomic sequencing to assess changes in the coding capacity of the gut microbiota during inflammation-associated dysbiosis [34]. This prior work suggested that bacterial genes encoding putative DH were enriched during colitis, a finding that prompted us to investigate in greater detail how microbial lactate metabolism changes during episodes of intestinal inflammation. Here, we discovered that during colitis, microbial lactate metabolism shifts towards increased utilization. In particular, the capacity to degrade lactate via respiratory lactate DHs is enhanced. Bacterial mutants lacking L-lactate DH activity exhibited decreased fitness in the inflamed gut. We also found that the increased availability of luminal lactate during non-infectious colitis is in part driven by inflammation-associated changes in epithelial metabolism. Collectively, our work suggests that the release of lactate by colonic epithelial cells during gut inflammation influences the metabolism of gut-associated microbial communities.

## Materials and methods

### Bacterial strains, plasmids, and primers

Bacterial strains and plasmids used in this study are listed in Table 1. All primers used for the study are listed in Table 2. Unless otherwise stated, all cultures were grown in lysogeny broth (LB) (10 g/L tryptone, 5 g/L yeast extract, 10 g/L sodium chloride) or on LB agar plates (10 g/L tryptone, 5 g/L yeast extract, 10 g/L sodium chloride, 15 g/L agar) under aerobic conditions at 37 °C. At times, the antibiotics Kanamycin (Kan), Carbenicillin (Carb), Nalidixic acid (Nal), and Ampicillin (Amp) were added to the growth media at concentrations of 100 mg/L, 100 mg/L, 50 mg/L, and 200 mg/L, respectively. All mutant strains (ST32, ST44, ST45, MW304) were generated by making clean deletions of the indicated gene. These deletions were generated by the following steps: All plasmids used for deletion of genes were generated using Gibson Assembly Cloning kit (new England Biolabs). Upstream and downstream regions, ~600 bp each, were amplified using the Q5 Hot Start High Fidelity DNA Polymerase (New England Biolabs) from the *E. coli* wild-type strain using primers generated by NEBuilder assembly tool as intended for Gibson Assembly. These fragments were introduced into SphI-linearized pGP706 by the Gibson Assembly protocol. These suicide plasmids were introduced into DH5α λ*pir* as a host. Once purified from the DH5α λ*pir* host via Plasmid Midi Kit (Qiagen), the plasmid was transformed into S17-1 λ*pir* which served as the donor strain for conjugation into the *E. coli* MP1 and Nissle 1917 strains. To select these strains after conjugation, the recipient strains contained the plasmid pSW172. Since pSW172 is a heat-sensitive plasmid, the conjugations were carried out at 30 °C. Once exconjugants were selected, they were grown at 37 °C in LB broth to cure pSW172, then plated onto sucrose media (5 % sucrose, 15 g/L agar, 8 g/L nutrient broth base) to select for bacteria in which the second crossover event had occurred. Then the deletions were confirmed using primers outside of the flanking regions used for the generating the plasmid. Electroporation was used to add plasmids to these strains as needed.

**Table 1:** Bacterial strains and plasmids used in this study

Strain or plasmid identifier	Description	Source
*E. coli* strains		
DH5α λ*pir*	F^−^*endA1 hsdR17* (r^−^m^+^) *supE44 thi-1 recA1 gyrA relA1* Δ(*lacZYA-argF*)*U189* φ*80lacZ*ΔM15 ﻿λ*pir*	[35]
S17-1 λ*pir*	*zxx::*RP4 2-(Tet^r^::Mu) (Kan^r^::Tn7) ﻿λ*pir*	[36]
MP1	*E. coli* wild-type strain	[37]
ST32	*E. coli* MP1 Δ*lldD*	This study
ST44	*E. coli* MP1 ﻿Δ*ykgEFG*	This study
ST45	*E. coli* MP1 Δ*dld*	This study
Nissle 1917	*E. coli* Nissle 1917	[38]
MW304	*E. coli* Nissle 1917 ﻿Δ*lldD*	This study
*S*. Tm strains		
IR715	Nalidixic acid-resistant derivative of *Salmonella enterica* serovar Typhimurium ATCC14028	[39]
AJB715	*S*. Tm IR715 ﻿Δ*phoN*::Kan^R^	[40]
CG6	*S*. Tm IR715 ﻿Δ*lldD*	[41]
Plasmids		
pGP706	*ori*(R6K) *mobRP4 sacRB* Kan^r^	[34]
pSW172	*ori(*R101) *repA101ts* Amp^r^	[23]
pST3	Upstream and downstream regions of *E. coli* MP1 *lldD* in pGP706	This study
pST4	Upstream and downstream regions of *E. coli* MP1 *ykgEFG* in pGP706	This study
pST6	Upstream and downstream regions of *E. coli* MP1 *dld* in pGP706	This study
pWSK29	*ori*(pSC101) *lacZ﻿α* Carb^R^	[42]
pWSK129	*ori*(pSC101) *lacZ﻿α* Kan^R^	[42]

**Table 2:** Oligonucleotides used in this study

Target/purpose	Sequence	Source
Primers for mutagenesis		
Deletion of *E. coli* MP1 *lldD*	5′-GCTTCTTCTAGAGGTACCGCATGGTGCTGCTCAGTCGACGTG-3′ 5′-CCCTTAAGCTTCATGCGTTTTTCTCCCTCG-3′ 5′- AAACGCATGAAGCTTAAGGGTTAGACGAATATC-3′ 5′-GGAGAGCTCGATATCGCATGTATGGTGATGGGATCTGAC-3′	This study
Confirmation of *E. coli* MP1 *lldD* deletion	5′-TGTCAGACGAGGTTGCC-3′ 5′-CAATCTGTGACGCTTGGC-3′	This study
Deletion of *E. coli* MP1 *dld*	5′-GCTTCTTCTAGAGGTACCGCATGCGACTGTTTTCACCGCATC-3′ 5′-TCCGTTATTATGTGGTGGCGAAAAAAATATC-3′ 5′-CGCCACCACATAATAACGGATGGCAGAG-3′ 5′-GGAGAGCTCGATATCGCATGCGCTGATGTCTTCAGAAAAC-3′	This study
Confirmation of *E. coli* MP1 *dld* deletion	5′-AAGCAGAGACACGCCCG-3′ 5′-AACAGCGAAATCAGCCG-3′	This study
Deletion of *E. coli* MP1 *ykgEFG*	5′-GCTTCTTCTAGAGGTACCGCATGGTGAATCATCTTTTCACAAG-3′ 5′-CATATCTCTCACTTCATGCCCATTTATG-3′ 5′-GGCATGAAGTGAGAGATATGTAGTCTGGAC-3′ 5′-GGAGAGCTCGATATCGCATGATGGTCAGGAGATAAGAG-3′	This study
Confirmation of *E. coli* MP1 *ykgEFG* deletion	5′-ATCGCTGAGTCAGTAGGC-3′ 5′-ATCAGACAACACCAGGC-3′	This study
Deletion of EcN *lldD*	5′-GCTTCTTCTAGAGGTACCGCATGGCCGATGATCCGGATTAC-3′ 5′-CGTCTAACCCGCGTTTTTCTCCCTCGAATG-3′ 5′-AGAAAAACGCGGGTTAGACGAATATCTGCTATCCTGC-3′ 5′-GGAGAGCTCGATATCGCATGCGGATACCCCAGCTGGCG-3′	This study
Confirmation of EcN *lldD* deletion	5′-AGGTGTGCTGCTCAGTCGAC-3′ 5′-GTGAAGTGGTGGAAGAAGCC-3′	This study
qPCR primers for *Mus Musculus*		
*Nos2*	5′-TTGGGTCTTGTTCACTCCACGG-3′ 5′-CCTCTTTCAGGTCACTTTGGTAGG-3′	[43]
*Tnf*	5′-AGCCAGGAGGGAGAACAGAAAC-3′ 5′-CCAGTGAGTGAAAGGGACAGAACC-3′	[34]
*Ifng*	5′-TCAAGTGGCATAGATGTGGAAGAA-3′ 5′-TGGCTCTGCAGGATTTTCATG-3′	[44]
*Gapdh*	5′-TGTAGACCATGTAGTTGAGGTCA-3′ 5′-AGGTCGGTGTGAACGGATTTG-3′	[44]

### Competitive growth assays in mucin broth

Mucin from porcine stomach type II (Sigma) was sterilized with 70% ethanol. The mixture was heated to 60 °C for 2 h, then incubated at room temperature overnight before drying in a vacuum centrifuge at 30 °C. Mucin broth was prepared by dissolving mucin in sterile autoclaved water, then adding magnesium sulfate (1 mM) and non-carbon E media. Sterile-filtered solutions of sodium nitrate, sodium L-lactate, and/or sodium D-lactate were added to reach final concentrations of 40, 20, and 20 mM respectively. Aliquots of 2 mL were incubated overnight in an anaerobic chamber at room temperature. Separate overnight cultures were inoculated with the designated strains and incubated while shaking aerobically in LB broth overnight. The mucin broth was then inoculated with 10^4^ CFU and grown either shaking aerobically or static in an anaerobic chamber for 16 h. Samples were then plated onto agar plates containing antibiotics selective for each strain. Recovery of each strain after 16 h was quantified by growth on agar plates. Competitive indices were calculated by dividing the ratio of wild-type over mutant bacteria recovered by the ratio of wild-type over mutant administered in the inoculum.

### Animal experiments

All experiments were conducted in accordance with the policies of the Institutional Animal Care and Use Committee at UT Southwestern. All mice were 6–20 weeks old with C57BL6/J genetic background and were bred under specific pathogen-free conditions in a barrier facility at UT Southwestern. All experiments include both male and female mice unless otherwise stated. All mice were on a 12-h light/dark cycle and consumed food and water ad libitum. For experiments using *Ccr2*^*−/−*^ and *Ldha*^ΔIEC^ mice, littermates were used as controls. *Il10*^*−/−*^ mice were initially purchased from Jackson Laboratory. *Ccr2*^*−/−*^ mice were purchased from Jackson Laboratory and crossed with wild-type C57BL6/J to generate littermate controls. For generation of the *Ldha*^ΔIEC^ mouse line, frozen embryos of *Ldha*^*tm1a*(EUCOMM)Wtsi^ mice were purchased from the European Mutant Mouse Archive and recovered in the Transgenic Technology Center at UT Southwestern. To remove the *LacZ* gene and neomycin resistance cassette, flanked by FRT sites, from the *Ldha* locus, the *Ldha*^*tm1a*(EUCOMM)Wtsi^ were crossed with mice that encode the FLPe recombinase. The progeny from this cross was then bred together to remove the FLPe recombinase gene. The resulting progeny was then mated with B6.Cg-Tg(Vil1-cre)997Gum/J mice, generating a conditional knockout of *Ldha* in the intestinal epithelium.

### Dextran sulfate sodium murine colitis model

Dextran sulfate sodium (DSS) (Alfa Aesar) was dissolved in water at a concentration of 1.5% and sterile filtered. DSS was placed in clean water bottles and administered to mice for 8 days. Mice received sterile water for another day before being euthanized.

### Streptomycin treatment

A solution of 200 mg/mL of streptomycin sulfate was prepared in water and sterile filtered. Then, 100 mL of the solution was administered to mice intragastrically for a total dose of 20 mg streptomycin per mouse. In experiments with both DSS and streptomycin treatment, the streptomycin was administered on day 6 of DSS treatment. In experiments with *Salmonella* infection, mice were treated with streptomycin 1 day before infection. Mice included in the same experiment but in a non-streptomycin group received 100 mL of autoclaved water intragastrically as a mock control.

### Piroxicam-accelerated colitis in *Il10*^*−/−*^ mice

*Il10*-deficient mice were given feed fortified with 100 ppm piroxicam (piroxicam from Sigma-Aldrich, fortified feed custom produced by Teklad) or mock-treated with standard laboratory feed for up to 10 days.

### Lactate and butyrate quantification by gas chromatography/mass spectrometry (GC/MS)

Intestinal content was collected into a tube containing sterile PBS. The tubes were vortexed for 2 min to resuspend the content in PBS. The samples were centrifuged for 15 min at 6000*g*, 4 °C, and the supernatant was collected. Deuterated lactate (CDN Isotopes) and deuterated butyrate (CDN Isotopes) were added to the aliquoted supernatant as internal standards. A standard curve was generated using sodium D,L-lactate, sodium butyrate, deuterated lactate, and deuterated butyrate dissolved in PBS. Samples were processed using two different extraction methods. For the experiments shown in Fig. 3F, G, samples and standards were dried in a vacuum centrifuge at 30 °C until only a dry pellet remained. Samples were resuspended in 100 μL of pyrimidine and sonicated. The pyrimidine resuspension was then incubated at 80 °C for 20 min. Then, 100 ﻿μL of *N*-Methyl-*N*-(tert-butyldimethylsilyl)trifluoroacetamide (MtBDSTFA) (Cerilliant) was added to each tube and incubated at 80 °C for 1 h. Tubes were then centrifuged at 22,000*g* for 5 min. Approximately 80 ﻿μL of the supernatant was transferred to a glass vial with a septum insert. For the experiments shown in Figs. 4D, E, 5A, B, 6A–D, and Fig. S3B, HCl was added to samples and standards to acidify the solution. Then, metabolites were extracted twice using ethyl acetate, added at a 1:1 ratio to each sample. The organic fractions were collected, combined, and anhydrous sodium sulfate salt was added to the mixture. The organic fraction was then vortexed and centrifuged at 22,000*g* for 2 min. After centrifugation, 50 ﻿μL of the supernatant was transferred to a clean tube, and 50 ﻿μL of MtBDSTFA (Cerilliant) was added to each tube. The tubes were vortexed to mix and then centrifuged at 22,000*g* for 30 s. The mixture was then incubated at 80 °C for 1 h. After incubation, the mixture was placed in a glass vial with a septum insert.

GC/MS analysis was performed with a Shimadzu TQ8040. The injection temperature was 250 °C and the injection split ratio was set to 1:100 with an injection volume of 1 μL. The oven temperature was set at 50 °C for 2 min, increasing to 100 °C at 20 °C per min and to 330 °C at 40 °C per min with a final hold at 330 °C for 3 min. The flow rate of the helium carrier gas (99.9999 % purity) was kept constant at a linear velocity of 50 cm/s. A 30 m × 0.25 mm × 0.25 μm Rtx-5Sil MS (Shimadzu) column was used. The interface temperature was 300 °C. The electron impact ion source temperature was 200 °C, with 70 V ionization voltage and 150 μA current. MtBDSTFA-derivatized butyrate (m/z of 145, 146, and 75) and deuterated butyrate (m/z of 152, 153, and 76) were quantitated in a single ion monitoring mode. MtBDSTFA-derivatized lactate (m/z 261>233, 261>189) and deuterated lactate (m/z 264>236, 264>189) were quantitated in multiple reaction monitoring mode. The target (quantitation) ion is underlined, all other fragments were used as reference (qualifier) ions. Concentrations were calculated based on an external standard. Calculated concentrations were adjusted by comparing the recovered internal standard to the known concentration of standard initially added to the sample. If the calculated concentration of the deuterated internal standard was less than 30 % of the initial concentration, the samples were excluded from analysis. In this study, samples with calculated concentrations below zero were marked as “below limit of quantification (LOQ).” To account for these values in calculations, the area of the detected peak was set equal to the lowest quantifiable peak for that run.

### Quantification of mRNA from intestinal tissue

Samples of whole tissue from the colon or cecum were harvested from euthanized mice, flash frozen, and stored at −80°C until RNA extraction. We extracted RNA using the TRI-Reagent method (MRC). For samples from experiments involving mice that were treated with DSS, mRNA was purified using Dynabeads mRNA DIRECT Kit (Invitrogen) to remove DSS and DNA contamination. For samples from experiments using *Il10*^*−/−*^ mice or *S*. Tm-infected animals, the extracted RNA was DNase treated using DNA-free DNase kit (Invitrogen). We prepared cDNA using MLV Reverse Transcriptase (Invitrogen) with TaqMan Reverse Transcription Reagents (Applied Biosystems). For Real-time PCR, we added the cDNA to SYBR green dye (Applied Biosystems) and primers from Table 2 and used QuantStudios RealTime 6 for analysis. Amplification of each target gene was normalized to amplification of the *Gapdh* housekeeping gene. If no amplification was detected by qRT-PCR (undefined C_T_) for any given target, the C_T_ value was set to 40 for calculations.

### Competitive colonization experiments with *E. coli*

Bacterial strains were grown overnight (16 h) in 100 mL of LB broth inoculated with three single colonies of the designated strain. Inoculum was prepared with a 1:1 ratio of wild-type strain to mutant with a final concentration of 10^10^ CFU/mL total bacteria. Wild-type and mutant bacteria harbored either pWSK29 or pWSK129 to facilitate recovery on selective media. The inoculum was diluted by ten-fold serial dilutions and plated. To colonize mice, 100 μL of the inoculum was administered by oral gavage (10^9^ CFU). In both DSS and *Il10*^*−/−*^ experiments, mice were colonized on day 7. After mice were euthanized, contents from the colon and cecum were collected separately in pre-weighed tubes containing sterile autoclaved PBS. The tubes were then vortexed for 2 min to resuspend the content in the PBS. Samples were serially diluted in PBS and plated on agar plates containing antibiotics selective for each strain. Recovery of each strain was measured by counting CFU of strains recovered on the corresponding antibiotic plate. CFU on plates were counted either by hand or with the ProtoCOL 3 colony counter (Synbiosis). Competitive indeces were calculated by dividing the ratio of wild-type over mutant bacteria recovered by the ratio of the wild-type strain over the mutant administered in the inoculum.

### Infection with *S.* Tm

*Salmonella* strains were grown overnight (16h) in 100 mL of LB broth inoculated with three single colonies of the designated strain. For single infection experiments, an inoculum was prepared with a single strain with a final concentration of 10^5^ CFU/mL total bacteria. For competitive infection experiments, an inoculum was prepared with a 1:1 ratio of Δ*phoN* (wild-type strain) to Δ﻿*lldD* mutant with a final concentration of 10^5^ CFU/mL total bacteria. The inoculum was diluted by serial dilutions and plated. To infect mice, 100 μL of the inoculum was administered by oral gavage. In these experiments, mice were infected 1 day after treatment with streptomycin (20 mg p.o.). Mice were euthanized 5 days after infection. After mice were euthanized, contents from the colon and cecum were collected separately in pre-weighed tubes containing sterile autoclaved PBS. The tubes were then vortexed for 2 min to resuspend the content in PBS. Samples were serially diluted in PBS and plated on agar plates containing the chromogenic substrate 5-bromo-4-chloro-3-indolyl phosphate (X-Phos) and nalidixic acid. Recovery of each strain was measured by counting CFU of blue vs. white strains recovered on the X-Phos-containing agar plate. CFU on plates were counted either by hand or with the ProtoCOL 3 colony counter (Synbiosis). Competitive indeces were calculated by dividing the ratio of wild-type bacteria over mutant bacteria recovered from the mouse by the ratio of the wild-type strain over the mutant administered in the inoculum.

### Metagenomics analysis

To profile changes in bacterial metabolic pathway abundance during gut inflammation, we reanalyzed a published metagenomic dataset of the DSS-induced murine colitis model (European Nucleotide Archive accession number PRJEB15095) [34, 45]. We performed adapter trimming, quality trimming, and quality filtering using the BBMap software suite (DOE Joint Genome Institute, Walnut Creek, CA). The quality of the processed reads was examined using FastQC (Babraham Bioinformatics), filtered against mouse genome (mm10) using Bowtie2 [46]. To evaluate the differential abundance of bacterial metabolic pathways, we mapped the processed reads to the UniRef90 database (UniProt Reference Clusters) [47] using the FMAP_mapping.pl command in the software package FMAP [48]. We then linked the Uniprot ID to KEGG database to identify the cognate KEGG orthology using FMAP_mapping.pl. The abundance of Uniprot and KEGG orthology was quantified using the FMAP_quantification.pl and FMAP_module.pl command. All analysis was done using the default parameters of the programs.

### Histopathology

Cecal tissue was fixed for 48 h in 10% buffered formalin phosphate (Thermo Fisher). Sections were cut from paraffin-embedded tissue and stained with hematoxylin and eosin. Sample slides were blinded and scored by a veterinary pathologist as described previously [23, 34].

### Statistical analysis

Statistical analysis was performed on GraphPad Prism 9. The specific statistical tests used to analyze differences in each experiment are noted in the figure legends. Mice that did not reach the experimental time point for animal welfare reasons and mice that were not colonized by one or both strains in the competitive colonization experiments were excluded from the analysis. Correlations between metabolites of interest and inflammation (combined histopathology scores) were calculated using the non-parametric Spearman’s rank correlation coefficient.

**Fig. 2:**
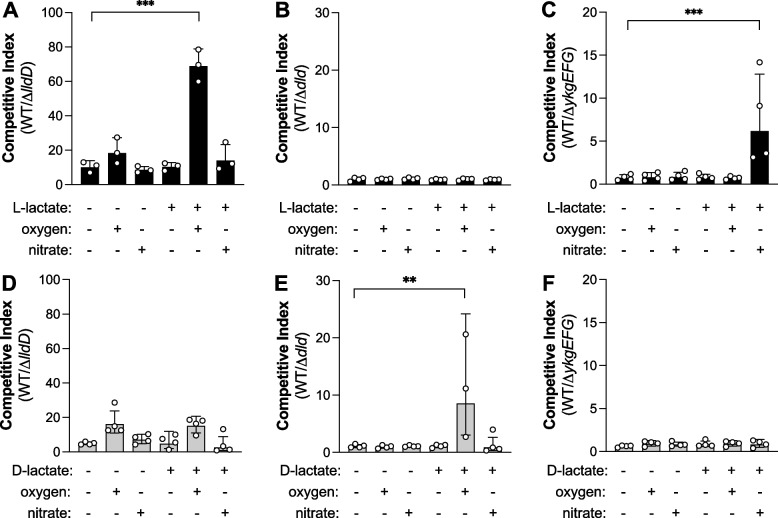
Utilization of lactate by *E. coli* in vitro*.* Mucin broth was inoculated with a 1:1 mixture of the *E. coli* MP1 wild-type and an isogenic strain lacking the indicated lactate dehydrogenase. Cultures were grown either aerobically (+ oxygen) or anaerobically (− oxygen). D-lactate, L-lactate, and nitrate were added as indicated. The competitive index represents the ratio of WT/mutant recovered after 16 h compared to the ratio of the inoculum. **A**–**C** L-lactate utilization by *E. coli* via the LldD enzyme (**A**), the Dld enzyme (**B**), and the YkgEFG enzyme (**C**). **D**–**F** Utilization of D-lactate by *E. coli* via the LldD enzyme (**D**), the Dld enzyme (**E**), and the YkgEFG enzyme (**F**). Columns represent geometric mean and error bars represent geometric standard deviation. Each dot represents one biological replicate. Statistical significance was assessed using a one-way ANOVA followed by Dunett’s multiple comparisons test on log-transformed data. **, *p*<0.01; ***, *p*<0.001

**Fig. 3:**
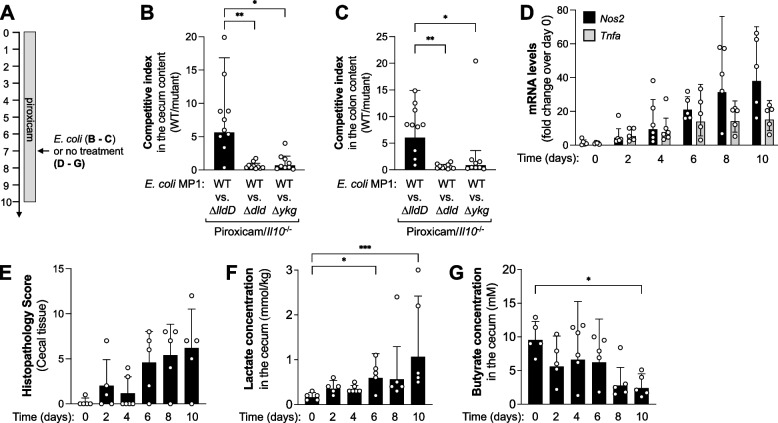
Lactate availability utilization by *E. coli* in a mouse model of non-infectious colitis. **A** Schematic of treatment regimens. **B**, **C ***Il10*-deficient mice received piroxicam-enriched feed to induce intestinal inflammation. After 7 days, mice were colonized with a 1:1 mixture of the MP1 *E. coli* wild-type strain (WT) and an isogenic mutant lacking the indicated lactate dehydrogenase. The abundance of the wild-type strain and the mutant in the cecal (**B**) and colon (**C**) content was determined 3 days after inoculation. The competitive index represents the ratio of WT and mutant recovered from the cecum content of mice at the end of the colitis treatment. **D**–**G** Groups of *Il10*-deficient mice received piroxicam-enriched feed and samples were collected at the indicated time points. **D** mRNA levels of *Nos2* (black bars) and *Tnfa* (gray bars)*,* normalized to *Gapdh*, in the cecal tissue as determined by RT-qPCR. **E** Combined histopathology scores describing the severity of inflammation in the cecal tissue. **F**, **G** Concentrations of lactate (**F**) and butyrate (**G**) in the cecum content measured by GC/MS. Columns and error bars represent the geometric mean and geometric standard deviation, respectively. In panel **E**, columns and error bars represent the mean and the standard deviation. Each dot reflects data from one animal. Statistical significance was determined using the Kruskal-Wallis test. *, *p*<0.05; **, *p*<0.01; ***, *p*<0.001

**Fig. 4:**
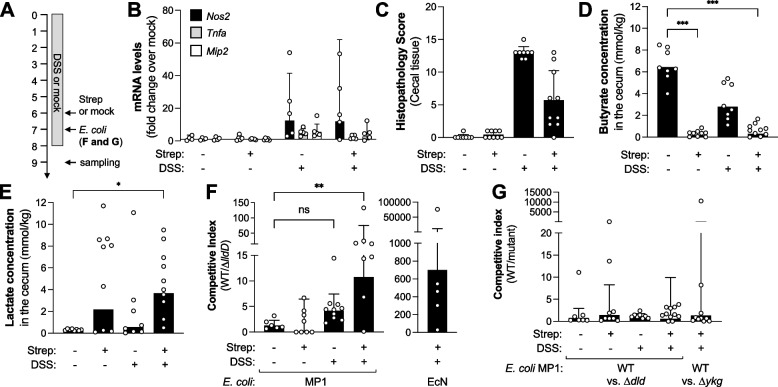
Effect of streptomycin and DSS treatment on intestinal inflammation and metabolite concentrations. **A** Schematic of treatment regimens. **B**–**E** Mice received 1.5% DSS in the drinking water or normal drinking water (mock). After 6 days of DSS treatment, mice were intragastrically administered either streptomycin (Strep) or water. Samples were obtained 9 days after start of the DSS treatment. **B** mRNA levels of iNOS (*Nos2;* black bars), TNF-a (*Tnfa;* gray bars), and MIP-2 (*Mip2;* white bars) in the cecal tissue as determined by RT-qPCR. **C** Combined histopathology scores describing the severity of cecal tissue damage. **D**, **E** Concentrations of butyrate (**D**) and lactate (**E**) in the cecum content measured by GC/MS. **F**, **G** Mice were treated with DSS and Strep as described above. Seven days after treatment start, mice were colonized with a 1:1 mixture of the *E. coli* wild-type strain (WT) and an isogenic mutant, as indicated. The competitive index represents the ratio of WT and mutant recovered from the cecum content. We assessed the contribution of LldD (**F**), Dld and YkgEFG (**G**). Columns and error bars represent the geometric mean and geometric standard deviation, respectively. In panel **C**, columns and error bars represent the mean and standard deviation, respectively. Each dot represents data from one animal. Statistical significance was determined using the Kruskal-Wallis test. *, *p*<0.05; ***, *p*<0.001

**Fig. 5:**
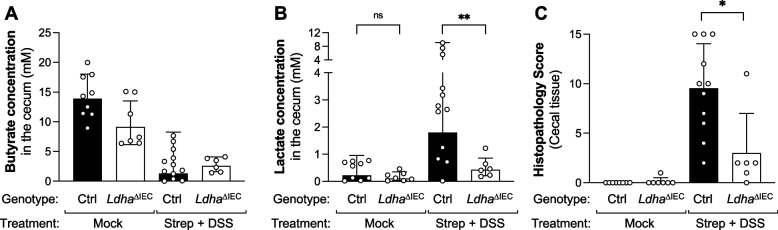
Contribution of epithelial lactate fermentation to host production of lactate during DSS colitis. **A**–**C** Groups of *Ldha*^ΔIEC^ mice and *Ldha*^*fl/fl*^ (Ctrl) littermates were treated with 1.5% DSS in the drinking water or received normal drinking water (mock). After 6 days of DSS treatment, mice were intragastrically administered either streptomycin or water. Samples were obtained 9 days after beginning of the DSS treatment. Luminal butyrate (**A**) and lactate (**B**) concentrations were measured in the cecum content by GC/MS. **C** Combined histopathology scores representing the severity of tissue damage. Columns represent geometric mean and error bars represent geometric standard deviation. Each dot represents one animal. Statistical significance was determined using a two-way ANOVA with Sidak’s multiple comparisons test. **, *p*<0.01; ns, not statically significant

**Fig. 6:**
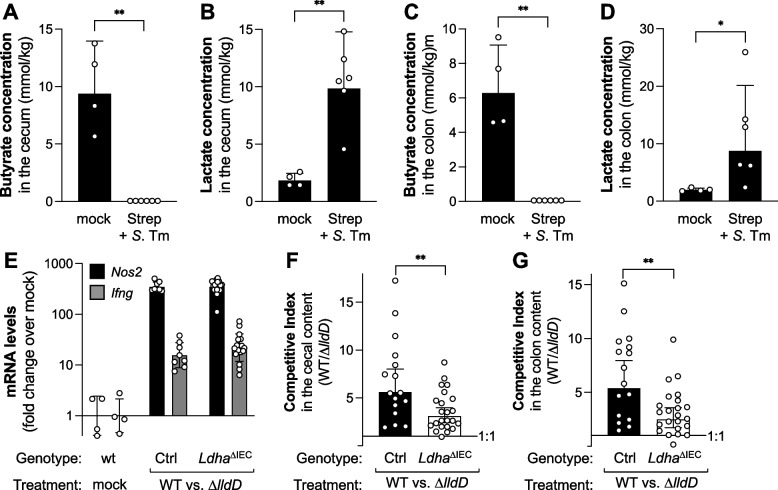
*Salmonella* consumes lactate generated by the intestinal epithelium. **A**–**D** Streptomycin-treated wild-type mice were infected with the *S.* Tm wild-type strain for 5 days. Mock-treated animals were used as controls. Butyrate (**A**) and lactate (**B**) concentrations in the colon content. Butyrate (**C**) and lactate (**D**) concentrations in the cecal content. **E**–**G** Wild-type mice (wt), *Ldha*^ΔIEC^ mice (*Ldha*^ΔIEC^), or *Ldha*^*fl/fl*^ (Ctrl) littermates were either mock-infected (mock) or infected with a 1:1 mixture of *S.* Tm AJB715 (WT) and the Δ*lldD mutant* for 5 days. **E** mRNA levels of iNOS (*Nos2*) and IFN-g (*Ifng*) in the cecal tissue, as determined by RT-qPCR. mRNA levels are normalized to Gapdh mRNA and represent the fold change over mock-treated wild-type mice. **F** Competitive index in the cecal content. **G** Competitive Index in the colon content. Columns represent geometric mean and error bars represent geometric standard deviation. Each dot represents one animal. Statistical significance was determined using a two-way ANOVA with Sidak’s multiple comparisons test. **, *p*<0.01; ns, not statically significant

The experiments in Fig. 2 are four independent replicates. The experiments in Fig. 3B and C were repeated twice for animals colonized with the wild-type vs. Δ﻿*dld* and wild-type vs. Δ﻿*ykgEFG* strains and three times for the wild-type vs. Δ﻿*lldD* strains. The experiments in Fig. 3D–G and Fig. S1 were performed once. The experiments shown in Fig. 4B–E and G, Figs. S2A and S3 were repeated twice. The experiments in Fig. 4F and Fig. S2B were repeated three times for the MP1 and once for the Nissle 1917 competition experiment. The experiments in Fig. 5 were repeated twice. The experiments in Fig. 6A–D were performed once and the experiments in Fig. 6E–G were repeated four times.

## Results

### Comparative metagenomics reveals a disease-associated shift in lactate metabolism

To better understand bacterial lactate metabolism in the context of colitis-associated dysbiosis, we re-analyzed a published dataset [34] using the FMAP pipeline [48] with a focus on pathways generating or consuming lactate (Fig. 1A, B). We found that genes encoding the fermentative L-lactate DH (*ldh*) decreased significantly in the microbiome of animals experiencing intestinal inflammation (Fig. 1C). The capacity to produce malolactic enzyme, which generates lactate through fermentation of malate, as well as the regulator of this pathway diminished during colitis, although this difference was not statistically significant (Fig. 1C). In contrast, several lactate utilization pathways were overrepresented in the context of colitis. Two (*ykgE*/*lldE*/*lutA* and *ykgF*/*lldF*/*lutB*) of the three genes encoding the heterotrimeric respiratory L-lactate DH YkgEFG/LldEFG/LutABC were enriched (Fig. 1C). We observed a similar trend for genes encoding the third subunit of this complex (*ykgG*/*lldG*/*lutC* and SO1518). Furthermore, genes encoding a lactate permease (*lldP*) [49, 50] were overrepresented in the disease condition implying increased uptake of extracellular lactate (Fig. 1C). Although not statistically significant, there was also a prominent increase in the abundance of genes encoding respiratory D- and L-lactate DHs, Dld, and LldD (also referred to as LctD in the literature), respectively. In contrast to the other respiratory lactate DHs, genes predicted to encode the FAD/FMN-dependent D-lactate DH (*dld-II*) decreased slightly. Overall, these findings suggest that the bacterial capacity to produce L-lactate from pyruvate (fermentative L-lactate DH) and malate (malolactic enzyme) diminishes during colitis, while the ability to take up and degrade lactate through respiratory lactate DHs increases. As such, we conclude that microbial lactate metabolism changes during experimentally induced colitis.

### Respiratory L-lactate DH enhances fitness of *E. coli* in mouse models of non-infectious colitis

We next sought to experimentally investigate bacterial lactate utilization by the respiratory lactate DHs YkgEFG/LldEFG/LutABC, LldD, and Dld. *E. coli*, a common member of the gut microbiota [51], expresses Dld, LldD, and LldP, allowing for the degradation of D- and L-lactate as well as transport, respectively [49, 52–55]. Furthermore, publicly available genome sequences predict that many *E. coli* strains encode a putative homolog (YkgEFG) of the LutABC system found in *Bacillus subtilis* and the LldEFG system from *Shewanella oneidensis* [56–58]. We therefore chose *E. coli* to assess bacterial lactate utilization in the murine gut and generated clean, unmarked deletions of *lldD*, *dld*, and *ykgEFG* in the murine commensal strain MP1 [37]. We then determined whether utilization of D- or L-lactate enhanced fitness under laboratory conditions in the presence of oxygen and nitrate as terminal electron acceptors (Fig. 2). Consistent with previous reports [49, 55], the *E. coli* wild-type strain outcompeted the *lldD* mutant under aerobic conditions in the presence of L-lactate, but not D-lactate (Fig. 2A, D). Similarly, the wild-type strain exhibited increased fitness compared to the isogenic *dld* mutant under aerobic conditions in the presence of only D-lactate (Fig. 2B, E). The mutant lacking the predicted YkgEFG lactate DH was recovered in similar numbers as the wild-type strain when cultured anaerobically in the presence of D- and L-lactate (Fig. 2C, F). When nitrate was added, the *ykgEFG* mutant exhibited a fitness defect in the presence of L-lactate, but not D-lactate. This outcome indicates that the *E. coli* YkgEFG lactate DH is indeed a respiratory L-lactate DH, akin to LutABC in *Bacillus* and LldEFG in *Shewanella* (Fig. 2C, F). Taken together, these experiments demonstrate that *E. coli* utilizes D- and L-lactate in a stereospecific manner to support growth and suggest that *E. coli* is a suitable model organism to explore the physiological functions of the respiratory lactate DHs, LldD, Dld, and YkgEFG.

The comparative metagenomics analysis predicted that lactate degradation through respiratory lactate DH increases during inflammation-associated dysbiosis. To test this prediction, we determined whether the respiratory lactate DHs enhance *E. coli* fitness in a murine model of colitis. *Il10*-deficient mice spontaneously develop cecitis (typhlitis) and colitis, a process that can be experimentally accelerated through oral administration of non-steroidal anti-inflammatory drugs such as piroxicam [59]. We treated *Il10*-deficient animals with 100 ppm piroxicam in their diet (Fig. 3A). After 7 days, we intragastrically inoculated groups of mice with an equal mixture of the MP1 wild-type strain and the *lldD* mutant, the wild-type strain and the *dld* mutant, or the wild-type strain and the *ykgEFG* mutant. Three days after inoculation, we determined colonization in the large intestinal content by plating on selective media. Under these experimental conditions, the wild-type strain outcompeted the *lldD* mutant in the cecum and colon content (Fig. 3B, C), indicating that *E. coli* utilizes L-lactate through its LldD lactate DH. In contrast, the *dld* and *ykgEFG* mutants exhibited no fitness defect in comparison to the wild-type strain (Fig. 3B, C). Several scenarios could account for the lack of a phenotype of the *dld* and *ykgEFG* mutants. It is possible that *E. coli* does not express YkgEFG or Dld under these experimental conditions, that lactate levels are insufficient for these enzymes to operate efficiently, or that lactate utilization through YkgEFG or Dld occurs but does not provide a quantifiable fitness advantage.

### The luminal lactate concentration increases in mouse models of inflammation and dysbiosis

Both the comparative metagenomics and the fitness assays performed with *E. coli* suggest that bacterial lactate utilization increases during inflammation. To better understand this phenomenon, we determined the availability of lactate as inflammation develops. mRNA levels of the inflammatory markers *Nos2*, encoding inducible nitric oxide synthase (iNOS), and *Tnfa*, encoding the pro-inflammatory cytokine tumor necrosis factor alpha (TNFa) (Fig. 3D) increased over the 10-day time course. Pathological changes to the cecal tissue rose correspondingly (Fig. 3E and Fig. S1A). Luminal lactate concentrations rose from 0.16 mmol/kg at the beginning of the treatment to approximately 1.1 mmol/kg at the final time point (Fig. 3F). Increased lactate concentrations correlated with inflammation (combined histopathology score; Spearman *r* = 0.59; *P* < 0.001). Butyrate levels decreased significantly over the course of the treatment (Fig. 3G), suggesting a disruption of fermentative microbiota metabolism. Lactate and butyrate concentrations were inversely correlated (Spearman *r* = −0.54; *P* < 0.01), as were butyrate concentrations and inflammation (Spearman *r* = −0.59; *P* < 0.001).

Dysbiosis does not only occur during intestinal inflammation. Oral antimicrobial therapy, for example, markedly disrupts the composition of the gut microbiota [60], and it is conceivable that lactate metabolism shifts under these conditions as well. To better understand the link between inflammation, dysbiosis, and increased lactate availability, we treated groups of mice with the aminoglycoside antibiotic streptomycin, a low dosage of DSS, or both (Fig. 4A). We assessed inflammation, lactate utilization by *E. coli*, and lactate availability, as well as butyrate concentrations as an indicator of gut microbiota function. Streptomycin administration had no discernable effect on inflammatory markers and pathology in the cecal tissue (Fig. 4B, C; Fig. S2A). Butyrate levels decreased drastically (Fig. 4D), implying a significant disruption of microbiota metabolism. Lactate availability increased moderately [24, 41] (Fig. 4E), but this was not significant when compared with mock-treated animals. LldD and Dld did not contribute to fitness of *E. coli* after streptomycin treatment (Fig. 4F, G; Fig. S2B and C). DSS treatment alone resulted in cecal inflammation, yet microbial butyrate output only decreased modestly and insignificantly when compared to mock-treated mice (Fig. 4B–E). In this setting, lactate levels did not rise significantly and LldD did not provide a significant fitness advantage to *E. coli* (Fig. 4F and Fig. S2B). Mice treated with both streptomycin and DSS exhibited mucosal inflammation, though the magnitude of inflammation was reduced when compared with DSS treatment alone (Fig. 4B, C) [61]. Butyrate availability was significantly decreased while lactate concentrations were significantly elevated, exceeding 3.6 mmol/kg (Fig. 4D, E). Only when animals were treated with both streptomycin and DSS did LldD provide a significant growth advantage to *E. coli* MP1 as well as the human *E. coli* isolate Nissle 1917 (Fig. 4F and Fig. S2B). YkgEFG and Dld appeared to be dispensable under these conditions (Fig. S2C). Collectively, these experiments demonstrate that microbial lactate consumption primarily occurs when intestinal inflammation coincides with impaired microbial butyrate production. Likewise, luminal lactate concentrations are highest when inflammation occurs simultaneously with low butyrate levels.

### Epithelial cells are a source of lactate during concomitant low-grade inflammation and microbiota dysbiosis

We next wanted to determine the source(s) of lactate production in colitis-associated dysbiosis. We found that the capacity of the microbiota to produce D-lactate DH (LdhA), a main pathway of bacterial lactate fermentation, was unchanged while the coding capacity to produce L-lactate through reduction of pyruvate (Ldh) or degradation of malate (malolactic enzyme) was likely diminished (Fig. 1). Furthermore, host-derived lactate supports growth of luminal *Salmonella enterica* serovar Typhimurium (*S*. Tm) in a murine model of Salmonellosis [41]. We thus hypothesized that the host might be a major source of lactate during non-infectious colitis.

During intestinal inflammation, circulating monocytes are recruited to the local tissue via chemokine (C-C motif) receptor 2 (CCR2), encoded by *Ccr2* [62–64]. At the site of inflammation, these monocytes mature into M1-type macrophages. Previous studies have shown that M1 macrophages typically exhibit an anaerobic glycolytic metabolism [65–67]. We considered the possibility that recruitment of M1 macrophages would drive an increase in luminal lactate during colitis. To test this hypothesis, we used *Ccr2*-deficient mice in which inflammatory macrophages cannot be recruited to the site of inflammation [63, 64]. We observed no difference between the concentration of luminal lactate in *Ccr2*-deficient mice compared to littermate controls, treated with streptomycin and low concentrations of DSS (Fig. S3A and B). Therefore, we concluded that infiltrating monocytes are unlikely to contribute to the accumulation of luminal lactate in a setting of antibiotic-induced microbiota perturbations and low-grade inflammation.

Under homeostatic conditions, the intestinal epithelium utilizes microbially produced butyrate as a preferred carbon source through β-oxidation [68]. When butyrate is not available, the intestinal epithelium undergoes a metabolic switch to an anaerobic glycolytic metabolism that generates L-lactate [69]. This switch is also controlled by regulatory T cells [24, 69]. In the piroxicam-accelerated *Il10* colitis model butyrate levels decrease as inflammation develops (Fig. 3). Similarly, the combined treatment with streptomycin and low-level DSS results in inflammation and a decrease of butyrate availability (Fig. 4), consistent with the idea that increased accumulation of lactate in these conditions could be a result of the intestinal epithelium switching from β-oxidation of butyrate to lactate fermentation. To test this hypothesis directly, we sought to generate mice which lacked lactate dehydrogenase in intestinal epithelial cells. Depending on the cell type, the isoenzymes of the mammalian lactate DH are either homotetramers of LDH-M or LDH-H subunits, or heterotetramers of both subunits [30]. LDH-M, encoded by *Ldha*, is the predominant subunit in the murine small and large intestine [70]. We therefore generated mice that lack expression of *Ldha* in intestinal epithelial cells (*Ldha*^ΔIEC^ mice) and treated *Ldha*^ΔIEC^ mice with streptomycin and DSS. In mock-treated animals, we observed no appreciable differences in luminal butyrate and lactate concentrations (Fig. 5A, B). Treatment of littermate controls with streptomycin and DSS increased lactate levels compared to mock-treatment (Fig. 5B). This increase in lactate concentrations was abrogated in *Ldha*^ΔIEC^ mice treated with streptomycin and DSS (Fig. 5B). These experiments indicate that lactate fermentation via the intestinal epithelial lactate dehydrogenase is contributing to the increase in luminal lactate during murine non-infectious colitis.

Inflammation-associated changes in the microbiota perpetuate mucosal immune responses and can instigate disease in genetically susceptibly hosts [11, 12]. We therefore determined whether the decreased availability of lactate in the *Ldha*^ΔIEC^ mice would result in changes in intestinal inflammation. Under homeostatic conditions, *Ldha*^ΔIEC^ mice did not exhibit any signs of inflammation. Curiously, when in the animals treated with DSS and streptomycin, a significant decrease in the inflammatory response (submucosal edema, epithelial damage, infiltration with inflammatory cells, and exudate) was apparent in *Ldha*^ΔIEC^ mice compared to littermate control mice (Fig. 5C and S3C).

### Epithelial cells are a source of lactate during *Salmonella* infection

To assess whether changes in host and microbial lactate metabolism are unique to non-infectious colitis or a general feature of inflammation, we turned to a mouse model of *Salmonella*-induced colitis (streptomycin-treated mouse model) [71]. This model recapitulates key aspects of human infection with non-typhoidal *Salmonella* strains, such as infiltration with neutrophils [71–73]. Infection with *S*. Tm decreases butyrate availability in the cecal and colonic content, as well as a prominent increase in lactate concentrations (Fig. 6A–D) [41]. We infected *Ldha*^ΔIEC^ mice and littermate controls with an equal mixture of the *S*. Tm wild-type strain and a mutant unable to utilize L-lactate (Δ*lldD*) [41]. In contrast to our finding that ablating epithelial LDH activity improves inflammation in a model of non-infectious colitis, we found no significant differences in cecal inflammation in *Salmonella*-infected *Ldha*^ΔIEC^ mice compared to littermate controls (Fig. 6E). This difference between infectious and non-infectious colitis could be due to the fact that in the infectious colitis model, inflammation is driven solely by *Salmonella* virulence factors [74]. Consistent with a previous report [41], the wild-type strain outcompeted the Δ*lldD* mutant (approximately 5-fold) in littermate controls (Fig. 6F, G). Importantly, the fitness advantage conferred by L-lactate utilization was significantly decreased in the *Ldha*^ΔIEC^ mice (Fig. 6F, G), indicating that *Salmonella* metabolizes epithelial-derived lactate during infection.

## Discussion

Due to its location at the host-microbiota interface, the intestinal epithelium constitutes a barrier against microbial intrusion. As such, the epithelium regulates the spatial distribution, function, and composition of the gut microbiota. For example, REG3γ, a soluble C-type lectin with antibacterial activity, is released by epithelial cells into the gut lumen where it contributes to the spatial separation of the microbiota and the host [75, 76]. Local depletion of molecular oxygen by the β-oxidation-based metabolism of colonocytes creates an anaerobic environment in the gut lumen and promotes colonization with obligate anaerobic bacteria [24, 77]. Reactive oxygen species produced by epithelial NADPH oxidase 1 (NOX1) during inflammation inhibit local colonization by catalase-negative bacteria and enhance the bloom of commensal and pathogenic Enterobacteriaceae family members [78, 79]. In our current studies, we illustrate another mechanism by which the epithelium shapes gut microbiota composition. We discovered that epithelial-derived lactate contributes to a spike of free lactate in the gut lumen in mouse models of non-infectious colitis. We found that the coding capacity of the gut microbiota to take up and degrade lactate increases during colitis. Furthermore, increased lactate consumption through the L-lactate dehydrogenase LldD promotes *E. coli* fitness in a mouse model of IBD. Our findings suggest that colonocyte-derived lactate impacts the metabolism of microbes in settings of infectious and non-infectious colitis, which expands our understanding of how the intestinal epithelium participates in host-microbiota interactions.

During homeostasis, microbiota-derived butyrate instructs colonocytes to perform β-oxidation. Facing dwindling butyrate availability during dysbiosis, colonocytes shift their metabolism to lactate fermentation [69]. The transcription factor peroxisome proliferator-activated receptor gamma (PPARγ) is involved in controlling colonocyte metabolism in response to butyrate. In mice lacking epithelial PPARγ, disturbances of colonocyte metabolism are only observed when an inflammatory cue is present [24]. In our current study, we found that increased lactate availability and *E. coli* lactate utilization only occurs when butyrate production, a key function of the microbiota, is perturbed and mucosal inflammation is present at the same time.

Transcription of *Ldha* in mammalian cells is regulated by several transcription factors, including the hypoxia-inducible factor (HIF) family of transcription factors [80]. Oxygen-sensing prolyl-hydroxylases regulate activity of HIF-1α and HIF-2α (EPAS1), the most studied members of this family, by controlling protein stability. Mice lacking HIF-2α in their intestinal epithelial cells exhibit reduced *Ldha* expression, decreased lactate levels in the intestinal lumen, altered bile acid availability, and changes in their gut microbiota [81]. Specifically, lactate-utilizing *Bacteroides vulgatus* were decreased in HIF-2α-deficient mice, raising the possibility that host-derived lactate might impact microbiota composition also under homeostatic conditions. Here, we show that depletion of LDH specifically in intestinal epithelial cells ablates an inflammation-associated spike in luminal lactate, providing direct evidence for the idea that lactate serves as a link between epithelial and microbial metabolism.

Ablation of LDH in epithelial cells decreased lactate levels in the cecal lumen of streptomycin and DSS-treated mice, but lactate levels were still slightly elevated compared to mock-treated animals. Furthermore, L-lactate utilization modestly enhances fitness of *S*. Tm in *Ldha*^ΔIEC^ mice, implying that *S*. Tm can access residual L-lactate in this setting. It is possible that, in addition to changes in epithelial metabolism, other mechanisms contribute to the observed increase in luminal lactate levels during colitis. Neutrophils use glycolysis and the pentose phosphate pathway to convert glucose to lactate [82], thus eliminating any potential competition between mitochondrial oxygen consumption and the generation of inflammatory reactive oxygen species. As such, neutrophils, and other infiltrating immune cells, could contribute to the lactate pool during inflammation [83]. Also, it has been proposed that small quantities of serum lactate can leak into the gut, where it supports propionate production by *Veillonella* [84]. Furthermore, it is possible that increased microbial production or decreased consumption of lactate could occur, though we did not find any evidence in our comparative metagenomics analysis to support this idea. However, due to limitations in the functional annotations in the databases used, not all possible reactions involving lactate were covered by our analysis. Future work is needed to resolve whether these and other potential mechanisms contribute to the increased availability of luminal lactate during episodes of inflammation.

One noteworthy finding of our study is that epithelial lactate metabolism is connected to the pathogenesis of non-infectious colitis in mice. Future studies will need to dissect whether epithelial lactate metabolism is directly modulating innate mucosal host responses or whether microbial (lactate) metabolism, downstream of epithelial lactate metabolism, is involved in mucosal inflammation. In either case, our finding that genetic ablation of LDH activity in epithelial cells alleviates inflammation suggests that host lactate metabolism is a potential target for novel intervention strategies that target the host-microbiota interface.

In IBD patients with active disease, functional and compositional changes in the gut microbiota occur alongside changes in the metabolite landscape [85]. Several studies have reported increased concentrations of fecal lactate in ulcerative colitis and possibly in a subset of Crohn’s disease patients compared to normal controls and quiescent IBD [86–91]. Montgomery et al. and Vernia et al. found that lactic acid levels correlate with severity of clinical disease [89, 90]. Similarly, a smaller study observed a trend for increased lactate levels in IBD patients and noted a positive correlation between fecal calprotectin and lactate levels [92]. Our experiments in the *Il10*-deficient mouse model of non-infectious colitis mirror this inflammation-associated increase in luminal lactate. Commonly discussed ideas to explain changes in lactate concentrations focus on microbial production and degradation. Our data suggest that a shift in host metabolism could also contribute to increased lactate levels in IBD. Consistent with that, mucosal secretion of L-lactate, the enantiomer produced by mammalian cells, but not D-lactate, occurs in patients with active ulcerative proctosigmoiditis [93].

## Conclusions

Collectively, our data suggest that during inflammation-associated dysbiosis, epithelial cells release lactate into the gut lumen where it impacts the metabolism of commensal gut microbes, such as *E. coli*, as well as pathogenic *Salmonella*. Our work highlights the importance of metabolic interactions between the epithelium and host-associated microbial communities in the context of inflammatory disease of the intestinal tract.

## Supplementary Information

**Additional file 1: Supplementary Figure S1.** Evaluation of pathological changes in the cecum in mouse colitis models. **Supplementary Figure S2.** Effect of streptomycin and DSS treatment on intestinal inflammation and *E. coli* lactate utilization. **Supplementary Figure S3.** Contribution of Macrophage Infiltration to Host Production of Lactate During DSS Colitis.

## Data Availability

The metagenomics dataset analyzed during the current study was published previously [34] and is available at the European Nucleotide Archive under the accession number PRJEB15095 (https://www.ebi.ac.uk/ena/browser/view/PRJEB15095). All other data generated or analyzed during this study are included in this published article and its supplementary information files.
