# Supplementary material for: Colonocyte-derived lactate promotes E. coli fitness in the context of inflammation-associated gut microbiota dysbiosis
Source: Microbiome. 2022 Nov 26;10:200. doi: 10.1186/s40168-022-01389-7 (PMC9701030; doi:10.1186/s40168-022-01389-7)
Supplement: Supplementary file 2 — Additional file 1: Supplementary Figure S1. Evaluation of pathological changes in the cecum in mouse colitis models. Supplementary Figure S2. Effect of streptomycin and DSS treatment on intestinal inflammation and E. coli lactate utilization. Supplementary Figure S3. Contribution of Macrophage Infiltration to Host Production of Lactate During DSS Colitis. [file 40168_2022_1389_MOESM1_ESM.pdf]

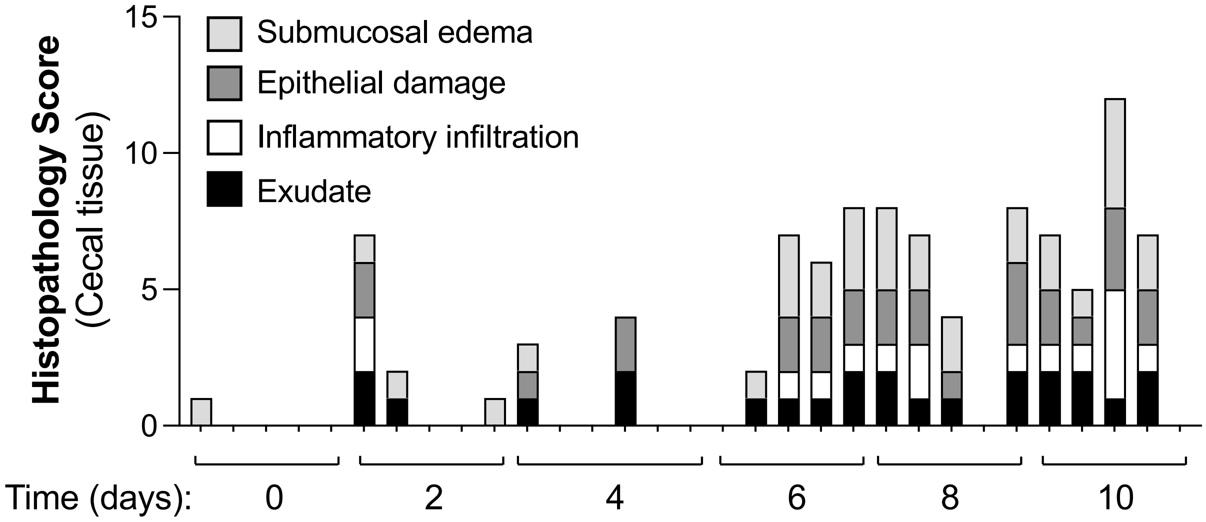

Supplementary Figure S1. Evaluation of pathological changes in the cecum in mouse colitis models. Related to Fig. 3. *Il10*-deficient mice were administered piroxicam-fortified feed and euthanized at the indicated time points. Sections of formalin-fixed cecal tissue were stained with hematoxylin and eosin, and the severity of submucosal edema (light gray), epithelial damage (dark gray), infiltration with inflammatory cells (white), and exudate (black) determined. Each column represents one animal.

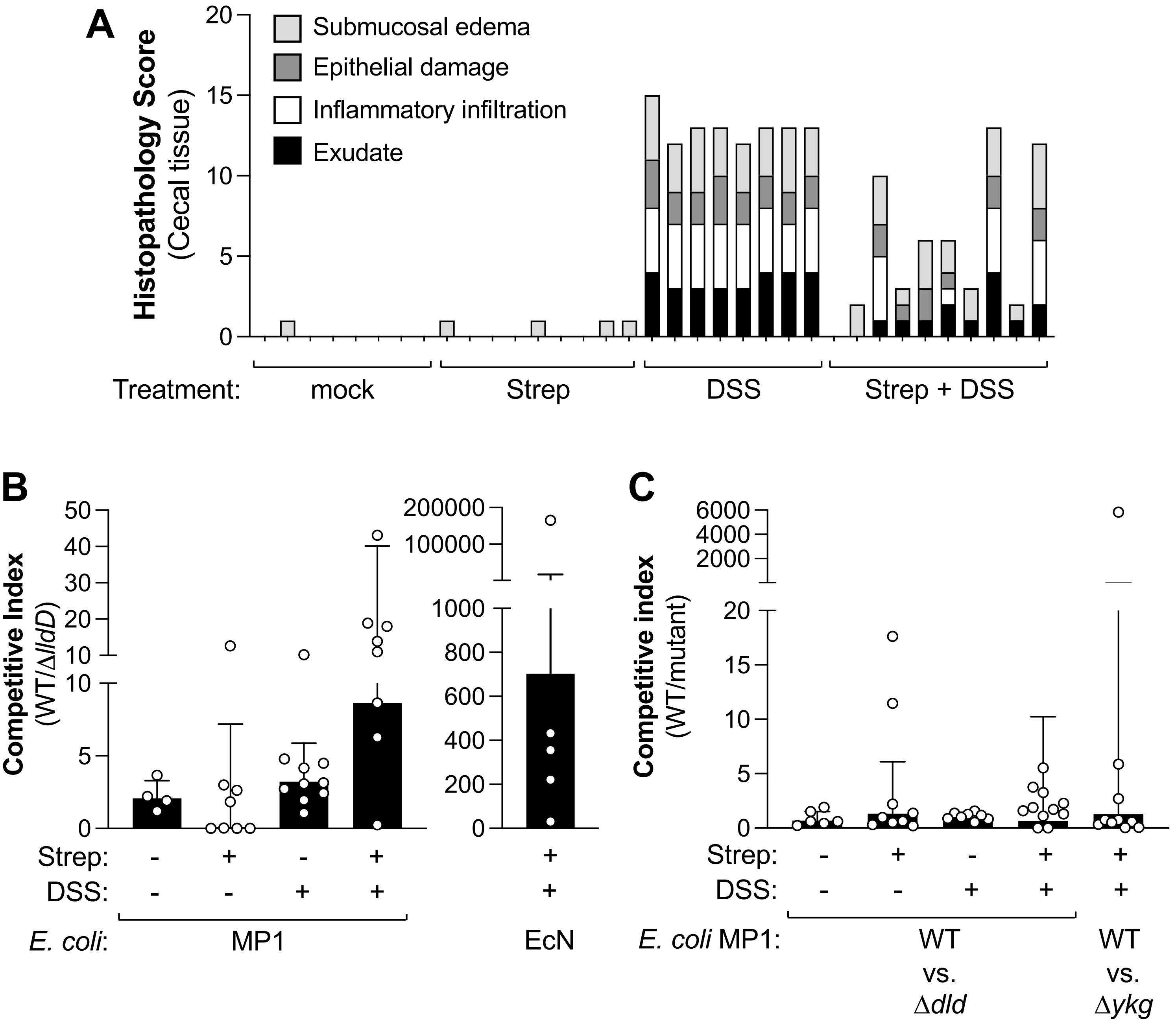

Supplementary Figure S2. Effect of streptomycin and DSS treatment on intestinal inflammation and *E. coli* lactate utilization. Related to Fig. 4. **(A)** Mice received 1.5% DSS in the drinking water or normal drinking water (mock). After 6 days of DSS treatment, mice were intragastrically administered either streptomycin (Strep) or water. Samples were obtained 9 days after begin of the DSS treatment. Sections of formalin-fixed cecal tissue were stained with hematoxylin and eosin, and the severity of submucosal edema (light gray), epithelial damage (dark gray), infiltration with inflammatory cells (white), and exudate (black) determined. Each column represents one animal. **(B - C)** Mice were treated with DSS and Strep as described above. Seven days after treatment start, mice were colonized with a 1:1 mixture of the *E. coli* wild-type strain (WT) and an isogenic mutant, as indicated. The competitive index represents the ratio of WT and mutant recovered from the cecum content. We assessed the contribution of LldD **(B)**, Dld and YkgEFG **(C)**. Columns and error bars represent the geometric mean and geometric standard deviation, respectively. Each dot represents data from one animal.

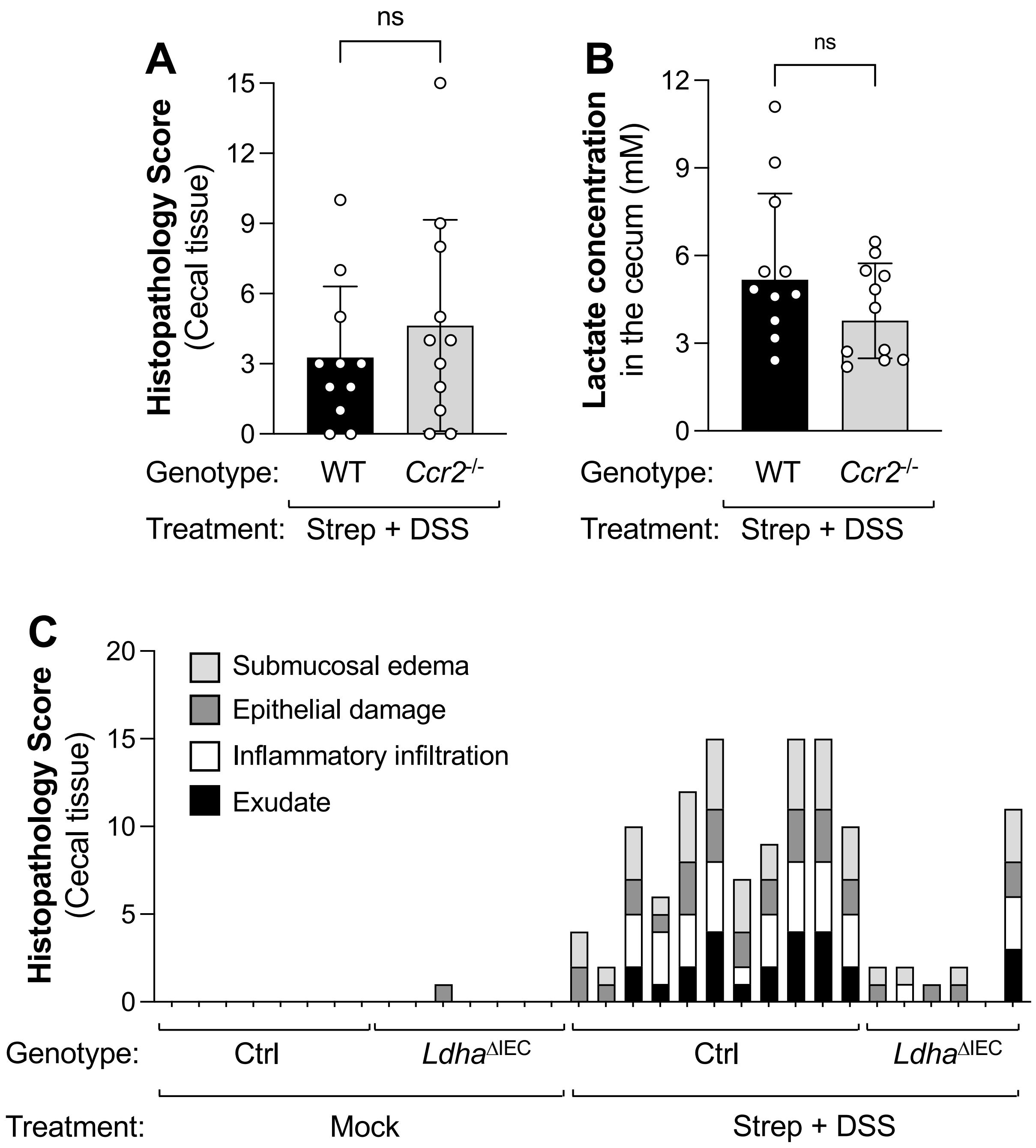

Supplementary Figure S3. Contribution of Macrophage Infiltration to Host Production of Lactate During DSS Colitis. Related to Fig. 5. Mice received 8 days of DSS treatment (1.5% DSS in drinking water). On day 6 of DSS treatment, mice were intragastrically administered streptomycin. **(A)** Luminal lactate measured in cecum content of *Ccr2*$^{-/-}$ mice or wild-type littermates **(B)** Luminal butyrate measured in cecum content of *Ccr2*$^{-/-}$ mice or wild type littermates. **(C)** Groups of *Ldha*$^{\Delta IEC}$ mice and *Ldha*$^{fl/fl}$ (Ctrl) littermates were treated with 1.5% DSS in the drinking water or received normal drinking water (mock). After 6 days of DSS treatment, mice were intragastrically administered either streptomycin or water. Samples were obtained 9 days after begin of the DSS treatment. Sections of formalin-fixed cecal tissue were stained with hematoxylin and eosin, and the severity of submucosal edema (light gray), epithelial damage (dark gray), infiltration with inflammatory cells (white), and exudate (black) determined. Each column represents one animal.

Columns represent geometric mean and error bars represent geometric standard deviation. Statistical significance was determined using the Mann-Whitney U test. *=$p<0.05$, **=$p<0.01$, ***$p<0.001$
